# The level of representation of irrelevant stimuli—Distractor–response binding within and between the senses

**DOI:** 10.3758/s13414-021-02249-6

**Published:** 2021-03-25

**Authors:** Ruth Laub, Simon Merz, Helena Dröschel, Christian Frings

**Affiliations:** Department of Cognitive Psychology, Trier University, Universitätsring 15, D-54296 Trier, Germany

**Keywords:** Distractor–response binding, Level of representation, Crossmodal binding

## Abstract

Binding theories assume that features of stimuli and executed responses can be integrated together in one event file (Hommel, *Visual Cognition, 5,* 183–216, 1998; Hommel, *Cognitive Sciences, 8,* 494–500, 2004). Every reencounter with one or more of the stored features leads to an automatic retrieval of the previously constructed event file and hence of the response—even the repetition of a task-irrelevant distractor stimulus can retrieve a previously encoded response. This so-called distractor–response binding effect is typically investigated using a sequential prime-probe design that allows the orthogonal variation of response relation (response repetition vs. resporrevertnse change) and distractor relation (distractor repetition vs. distractor change), while probe response times and error rates are measured as dependent variable. Previous research has shown that task-relevant stimuli can be represented at different levels (e.g., perceptual and conceptual; see Henson et al., *Trends in Cognitive Sciences*, *18*, 376–384, 2014), yet it is not clear at which level of representation distractor stimuli are processed. In the present study, we focused on the level of representation of response-irrelevant distractor stimuli. To this end, a crossmodal distractor–response binding paradigm was used that enables the differentiation between the perceptual and conceptual representation of the distractor by allowing the systematic repetition and change of conceptual distractor features independent of perceptual repetitions. The results suggest that the repetition of perceptual distractor features is indispensable for the initiation of the retrieval process while the sole repetition of conceptual distractor features is not sufficient to start the retrieval process.

In daily life, we act in an environment that is continuously confronting us with many different objects, offering many different action opportunities. Thus, for the successful execution of actions, action control is indispensable. In the past few decades, one important mechanism in action control that was identified is the binding of stimuli and responses. According to the theory of event coding (TEC; Hommel, 2004; Hommel et al., 2001), perceived stimulus or object features (such as shape, color, and location) and executed responses are encoded together in one short-lived memory trace, called an event file. Furthermore, a reencounter with one or more of the stored features leads to the automatic retrieval of the previously constructed event file and thus of the response features, thereby influencing current actions. If all integrated features and the response are repeated, response execution is facilitated, while performance is impeded if only some of the stimulus or response features are repeated (Hommel, 1998, 2005). This so-called binding effect indicates that feature repetitions have a direct impact on behavior (see Frings et al., 2020, for a recent framework on integration and retrieval effects in action control). Interestingly, binding effects have not only been observed for features belonging to the response-relevant target stimulus but also for response-irrelevant distractor stimuli that accompany the target. The distractor–response binding effect showed that the repetition of irrelevant distractor stimuli can just as well retrieve a previously constructed event file and consequently also the previously integrated response (Frings et al., 2007; Rothermund et al., 2005). The distractor–response binding effect is typically investigated using a sequential prime–probe design, which allows the orthogonal variation of response relation (i.e., whether the response is repeated or changed from prime to probe) and distractor relation (i.e., whether the distractor is repeated or changed from prime to probe). The distractor–response binding effect then arises in the interaction of the factors distractor relation and response relation: Repeating a distractor in the probe enhances performance in case of response repetition, while performance is impaired if a different response is required in the probe. More precisely, distractor repetitions should facilitate performance if the response is repeated as well, since the repeated distractor in the probe retrieves the previously integrated prime episode including the response, which is compatible with the demanded probe response. In contrast, distractor repetitions should hamper performance if the response is changed, since the repeated distractor in the probe retrieves the incompatible prime response. The difference of distractor repetitions in response repetition and change trials constitutes the distractor–response binding effect. Thus the distractor–response binding effect can be interpreted as the amount of response interference or facilitation due to distractor-based retrieval.

One of the interesting research questions in the binding literature is whether stimulus features can be encoded and retrieved at different levels of representation (e.g., perceptual or abstract/semantic; Henson et al., 2014; Horner & Henson, 2011). This question arises not only for the representation of response-relevant target stimuli but also for the representation of response-irrelevant distractor stimuli (that is, in the context of the present study, distractors that are never mapped to a response and thus are completely task irrelevant). Most studies indicate a direct link between perception and action in the context of stimulus–response bindings. That is, the repetition of perceptually identical target features can retrieve a previously integrated response (e.g., Hommel, 2005). However, there is also evidence suggesting that the repetition of a semantic target feature can retrieve a previously encoded episode (e.g., Henson et al., 2014; Horner & Henson, 2011).

With regard to the level of representation of response-irrelevant distractor stimuli, similar evidence was found. For instance, the repetition of perceptual distractor features can retrieve a previously encoded response, without a representation and repetition of the distractor at a conceptual level (e.g., Laub & Frings, 2020). Moreover, previous research suggested that irrelevant stimuli can even be represented at a conceptual level (Frings et al., 2013; Wesslein & Frings, 2020). Frings and colleagues randomly switched the modality of distractor and target stimuli (sounds versus pictures of animals) in a distractor–response binding paradigm. The authors observed that the repetition of a distractor at a conceptual level could retrieve a previously executed response, although the perceptual distractor features changed (i.e., repeating the identity of the animal in a different modality). For instance, if the prime distractor was the picture of a frog and the probe distractor was the sound of a frog, the probe distractor could retrieve the prime response associated with the concept frog. The authors interpreted their results as evidence for distractor-based retrieval at a conceptual level. Wesslein and Frings (2020) observed comparable results in another distractor-based task—namely, negative priming. The negative priming effect refers to the phenomenon that when a to-be-ignored distractor stimulus in the prime is repeated as a to-be-selected target in the subsequent probe, performance is impaired (for reviews, see Frings et al., 2015; Tipper, 2001). In line with an inhibition view (Houghton & Tipper, 1994; Houghton et al., 1996), it is assumed that actively ignoring a distractor leads to some kind of inhibited representation of that distractor. This inhibition lingers from trial *n* − 1 to trial *n*, leading to worse performance if one has to respond to a still inhibited stimulus (but also see Neill et al., 1992, for an episodic retrieval view).

Yet in the study by Frings et al. (2013) and Wesslein and Frings (2020), both modalities were response relevant in half of the trials. As shown by Zmigrod and Hommel (2010), the specification of a modality as task relevant leads to the construction of an attentional set that weights stimulus features from the same sensory modality to a higher degree, thus leading to a stronger impact of stimuli from this modality (see also Jensen et al., 2019a). Consequently, the distractor stimuli in the experiments by Frings et al. (2013) should have received ample attention due to the relevance of the modality and thus are likely processed to a higher degree (i.e., up to a semantic/conceptual level; see Laub & Frings, 2020; Singh et al., 2018). Even more, the distractor stimuli were drawn from the same stimulus set as the targets and consequently were mapped to specific responses. Thus, the retrieval process may be initiated by the repetition of response features rather than by the repetition of conceptual features (see Singh et al., 2019, for a similar discussion). In sum, the existing evidence is not clear about whether distractor-based retrieval can actually operate at the level of conceptual distractor representations, without additional assumptions like an attentional set that comprises the distractor modality.

## The present study

The present study was specifically designed to investigate the level of representation of response-irrelevant distractor stimuli. Therefore, the experiment needed to meet different specific criteria. First, the distractors and their features needed to be completely irrelevant to the task. Second, it was necessary to choose distractor stimuli with clearly distinguishable perceptual and conceptual features. Third, the experiment needed to enable a repetition of conceptual distractor features independent of the repetition of perceptual distractor features.

To meet these criteria, we used a crossmodal distractor–response binding paradigm and developed a task context in which the distractor identities and the distractor modalities were never task relevant. To accomplish this, three different modalities were used in the present experiment. Targets were always presented to vision, whereas distractors could be presented to audition or touch. Thus, the distractor modality could either be repeated or changed from prime to probe. Additionally, different rhythms were used as distractor stimuli, whereas different colors were used as target stimuli. As previous studies have shown (e.g., Frings & Spence, 2010; Wesslein & Frings, 2020), the use of rhythms is ideal for this kind of investigation, since a rhythm can be defined as temporal pattern of signal-present and signal-absent events. This temporal pattern constitutes the stimulus identity, or (as it is called in the present study) the concept/conceptual distractor feature. Importantly, this temporal pattern can be presented in different sensory modalities. We call the particular modality the rhythm is presented to the percept/perceptual distractor feature in the present study. Previous studies have already shown that the identity of a stimulus and the modality of a stimulus can be independently varied and investigated when presenting rhythms to different modalities. For instance, Frings and Spence (2010) used a crossmodal congruency task and orthogonally varied the stimulus identity and the stimulus modality of the presented target and distractor rhythm. The authors found a significant crossmodal congruency effect, which suggests crossmodal interactions in the processing of the stimulus identity.

In this trimodal design, conceptual distractor features (i.e., the distractor identity/the specific rhythm) can be repeated without a repetition of perceptual distractor features (i.e., the distractor modality), while both (the distractor identity and the distractor modality) are never task relevant. If crossmodal distractor-based retrieval is observed in this task (i.e., distractor-based retrieval due to the sole repetition of conceptual features), this suggests that a distractor is represented at a conceptual level, since there is no repetition of the distractor at a perceptual level. If distractor-based retrieval is, however, only observed if the perceptual distractor features are repeated, this suggests that the distractor is represented at a perceptual level.

Different assumptions about the level of representation of a distractor stimulus can be drawn. On one side, it is possible that a distractor stimulus is only represented at a perceptual level (in a modality-specific way)—that is, the identity of the distractor stimulus is encoded in a specific modality (i.e., the percept). Thus, one would expect distractor-based retrieval only if the identity (i.e., in our study, the rhythm) is repeated in the specific modality (i.e., the percept). In this case, the representation of the stimulus is located at a perceptual (modal) level and the repetition of perceptual distractor features is indispensable for the initiation of the retrieval process. In line with this assumption, we would expect to find distractor-based retrieval (as indicated by the interaction of response relation and distractor relation) solely if the distractor modality is repeated from prime to probe. This would be confirmed by a significant three-way interaction of response relation, distractor relation, and modality relation, evidencing that the distractor–response binding effect is only observed if the distractor modality is repeated.

On the other side, it could be assumed that the distractor is represented in a more abstract way, independent of the specific modality—that is, at a conceptual level. In this case, the repetition of the stimulus identity in a different modality (i.e., the repetition of the distractor identity without repeating the concrete percept) can initiate the retrieval process (crossmodal distractor-based retrieval). Thus, the representation of the stimulus would be located at an abstract (amodal) or conceptual level. According to this assumption, we would expect to find a significant distractor–response binding effect independent of whether the distractor modality (the perceptual distractor feature) is repeated or changed from prime to probe. Statistically, this would be indicated by a significant two-way interaction of response relation and distractor relation that is not further specified by modality relation.

## Method

### Participants

Forty students (34 females) from University of Trier took part in the experiment. The median age of the participants was 21 years (range: 18–47 years). All participants reported normal or corrected-to-normal vision. An a priori calculation of the sample size indicated that a minimum of 38 participants is needed to observe a middle-sized effect (Cohen’s *f* = 0.25), assuming a power of 1 − ß = .85, an alpha of .05, and a medium correlation of 0.5 (G*Power 3.1.9.2; Faul et al., 2007). We decided to collect data from 40 students to compensate for possible dropouts. All participants took part in exchange for partial course credit.

### Design

The experimental design consisted of three within-subjects factors—namely, response relation (response repetition vs. response change), distractor relation (distractor repetition vs. distractor change), and modality relation (modality repetition vs. modality change).

### Materials and apparatus

The experiment was conducted using E-Prime software (Version 2.0), and the data were analyzed with IBM SPSS Statistics (Version 23). Instructions were shown on a standard TFT screen (60 Hz; 1,920 × 1,200 pixels). Viewing distance was approximately 70 cm, and participants responded using a standard mouse connected via USB port.

**Fig. 1:**
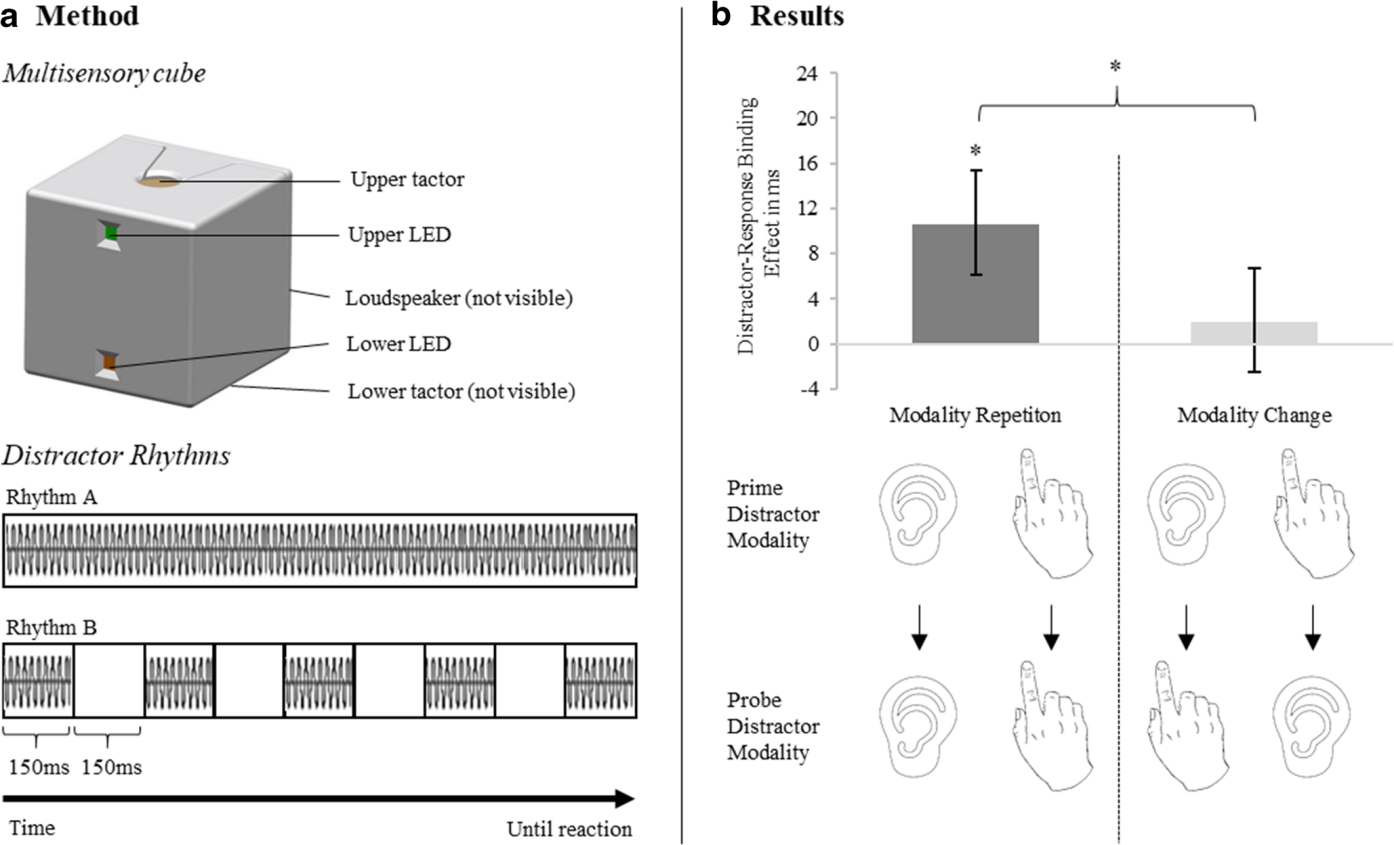
Experimental setup and results of the experiment. **a** Illustration of the multisensory cube with its features (upper panel) and the Rhythm A and B used as auditor or tactile distractors (lower panel). **b** Distractor-response binding effects in milliseconds as a function of modality relation. Error bars depict standard error of the means. * *p* < .05

All stimuli were presented on a custom-made cube (see Fig. 1a) (70 mm^3^; see Merz et al., 2019, for a detailed description of the custom-made cube). On the front side of this cube, two LED lights are attached. The backside is equipped with an integrated loudspeaker and one vibrotactile tactor (Model C-2, Engineering Acoustic, Inc.; controlled via the serial interface) was located in the center of the top as well as bottom side of the cube. The upper LED as well as the upper tactor were used for the experiment. Four target colors—namely red (RGB: 255, 0, 0), green (RGB: 0, 255, 0), blue (RGB: 0, 0, 255), and yellow (RGB: 255, 255, 0)—were used, and two tactile (~270 Hz, ~ 70 μm peak-to-peak amplitude) and two corresponding auditory distractor rhythms (1670 Hz, ~69 dB SPL) were presented (see Fig. 1a). Rhythm A was either a continuous tone or a continuous vibration, which was presented until the participant responded. Rhythm B was a compound of alternating vibrations/tones (150 ms) and blank interstimulus intervals (150 ms) that was presented until the response was executed.

In order that the tactile stimuli are only perceived tactually and that the sound elicited by the tactors (a 270-Hz sound) would not be heard by the participant, brown noise (~76 dB SPL) was presented during the whole experiment over two loudspeakers (part of the Wavemaster MX3+ 2.1 channel stereo sound system, Hightech Media Components, Germany) on the left and right of the screen. Due to presenting brown noise in a frequency that masked the low-frequency sound elicited by the tactor, auditory stimuli with a higher frequency had to be used in the experiment. Thus, auditory and tactile distractor stimuli were presented in different frequencies. However, the pattern of signal-present and signal-absent events, which represent the specific rhythm, is the same independent of whether it is present with a high or a low frequency.

### Procedure

Participants were tested individually in a completely darkened room (no daylight, black furniture) to exclude all sources of sensations except the stimuli. Experimental instructions were presented on-screen. Participants were asked to hold the custom-made cube with their left hand, place their index finger on the upper tactor, and hold it centrally in front of them just below the screen. The right hand was used to operate the mouse. Participants were instructed to respond to the color of the upper LED as rapidly and accurately as possible by pressing the respective mouse key. Two colors were mapped to one key. Half the participants respond to the green and blue light with the right key and to the red and yellow light with the left key, the other half received the reversed mapping. Furthermore, subjects were instructed to ignore the tactile and auditory rhythms (i.e., the distractors), which were presented simultaneously with the light.

A single trial consisted of the following events: Each trial started with a 500-ms blank interval with no stimulus presentation, immediately followed by the prime, which consisted of the response-relevant light-up of one of the four possible visual stimuli and the simultaneous presentation of one of the two possible distractor rhythms. The particular rhythm was presented either as a sound or as a vibration. The prime lasted until participants responded by pressing one of the two mouse keys. In case of an error, an error display appeared for 1,000 ms. Then the cube turned blank for 500 ms, followed by the probe stimuli. Again, one of the four visual stimuli and either a tactile or auditory distractor rhythm were presented until the participants responded. After that, a 500-ms blank interval followed, and then the next trial started. Both the target and the distractor identity could be repeated or changed between prime and probe.

In response repetition trials, the color of the target in the prime and probe always indicated the same response. In half of the response repetition trials, the target identity is repeated; in the other half, it changed. In response change trials, the color of the target in the prime and probe always indicated different responses. In distractor repetition trials, the rhythm of the distractor in the prime and probe was repeated. In distractor change trials, the rhythm of the distractor changed from prime to probe. In modality repetition trials, the modality of the distractor rhythm remained the same in the prime and the probe (i.e., the prime and the probe distractors were both either tactile or auditory). In modality change trials, the modality of the distractor changed from prime to probe (i.e., the prime and the probe distractor were always presented in different modalities). The orthogonal variation of the factors response relation, distractor relation, and modality relation led to eight conditions. Each trial condition was realized in 96 trials, resulting overall in 768 trials. Every 80 trials, participants had the possibility to take a break. Prior to the experimental block, all participants had to work through a practice block consisting of 20 trails. The sole difference in the practice block was that participants received feedback for both incorrect and correct responses, while in the experimental block, they only received feedback for incorrect responses. All trials were presented in random order.

### Analysis

The distractor–response binding effect arises in the interaction of response relation and distractor relation in the statistical analyses. Furthermore, the distractor–response binding effect can be computed as the distractor repetition effect in response repetition trials minus the distractor repetition effect in response change trials. The distractor–response binding effect is thus calculated using the following formula: (response repetition / distractor change − response repetition / distractor repetition) − (response change / distractor change − response change / distractor repetition) for both RTs and error rates. While all analyses were calculated with the raw RT and error data, the distractor–response binding effect was calculated with the formula for the illustration of the distractor–response binding effect in Fig. 1b.

## Results

Only trials with correct responses to both prime and probe were included in the analysis. Trials shorter than 100 ms or longer than 1.5 interquartile ranges above the third quartile of the reaction time (RT) distribution of each participant (Tukey, 1977) were not included in analysis. This resulted in a total of 13.87% of the data being excluded from the RT analysis: 5.03% of the trials were excluded because of erroneous responses in the prime, 4.45% of the trials were excluded because of erroneous responses in the probe, and 4.39% due to the RT outlier criteria. Two subjects made more than 10% errors in the probe and in addition had more than 20% missing trials due to errors and outlier criteria, and were therefore excluded from the analyses.^1^ See Table 1 for mean RTs and probe error rates.

**Table 1:** Mean reaction times in milliseconds and error rates in percentages (standard deviation in parentheses) of probe responses as a function of response relation, distractor relation, and modality relation

		Modality relation			
		Modality repetition		Modality change	
		Response repetition	Response change	Response repetition	Response change
Distractor repetition	RT (*SD*)	481 (46)	557 (53)	509 (51)	551 (60)
	Error (*SD*)	4.6 (2.5)	6.0 (3.8)	6.0 (3.5)	3.2 (2.4)
Distractor change	RT (*SD*)	488 (53)	554 (51)	509 (55)	549 (55)
	Error (*SD*)	4.3 (3.1)	5.0 (2.7)	5.2 (3.3)	3.6 (2.5)

In a 2 (response relation: response repetition vs. response change) × 2 (distractor relation: distractor repetition vs. distractor change) × 2 (modality relation: modality repetition vs. modality change) analysis of variance (ANOVA) on probe RTs, the main effect of response relation, *F*(1, 37) = 247.31, *p* < .001, η_p_^2^ = .87, was significant. Participants responded faster if the response was repeated (*M* = 497 ms, *SD* = 51 ms) than if the response was changed (*M* = 553 ms, *SD* = 55 ms). The main effect of modality relation, *F*(1, 37) = 33.01, *p* < .001, η_p_^2^ = .47, was significant, too. Responses were faster if the modality was repeated from prime to probe (*M* = 520 ms, *SD* = 51 ms) than if the modality changed (*M* = 529 ms, *SD* = 55 ms). The main effect of distractor relation did not reach significance, *F*(1, 37) < 1, *p* = .621, η_p_^2^ < .01.

**Fig. 2:**
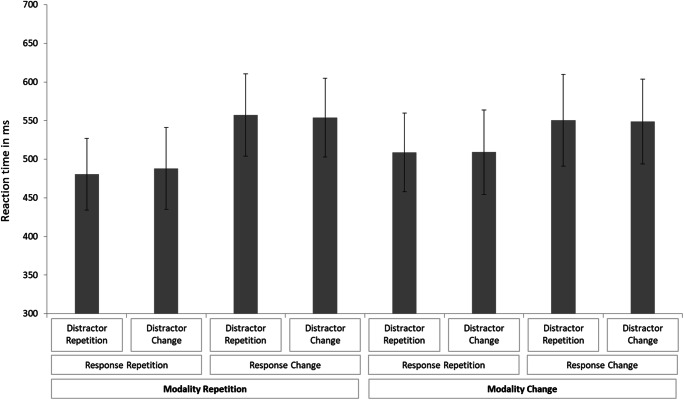
Probe reaction times as a function of distractor relation, response relation, and modality relation. Error bars depict standard deviation of the means

The interaction between response relation and distractor relation was not significant, *F*(1, 37) = 3.90, *p* = .056, η_p_^2^ = .10. However, the descriptive trend indicated a general distractor–response binding effect.^2^ Most importantly, the three-way interaction between modality relation, response relation, and distractor relation was significant, *F*(1, 37) = 5.56, *p* = .024, η_p_^2^ = .13. 3 The distractor–response binding effect differed between trials with modality repetition and trials with modality change. Post hoc analysis indicated that the distractor–response binding effect was only significant for modality repetitions, *t*(37) = 3.06, *p* = .004, *d* = 0.50 (BF_10_ = 8.85, which can be considered as substantial evidence for the alternative hypothesis; see Wagenmakers et al., 2011), but not for modality changes, *t*(37) = 0.51, *p* = .614, *d* = 0.08 (BF_01_ = 5.07, which can be considered as substantial evidence for the null hypothesis; see Wagenmakers et al., 2011; see Fig. 1b; see also Fig. 2 in the Appendix for a more detailed presentation of the reaction times in the different conditions).^4^

For the sake of completeness, the interaction of modality relation and response relation was significant, *F*(1, 37) = 73.69, *p* < .001, η_p_^2^ = .67. The RT benefit due to response repetition (as compared with response change) was greater if the modality of the distractor was repeated (*M* = 74 ms, *SD* = 24 ms) than if the modality changed (*M* = 46 ms, *SD* = 31 ms). The interaction of modality relation and distractor relation did not reach significance, *F*(1, 37) = 1.30, *p* = .262, η_p_^2^ = .03.

The same ANOVA was conducted on probe error rates yielding a nonsignificant distractor–response binding effect—that is, the interaction between response relation and distractor relation—did not reach significance, *F*(1, 37) = 0.22, *p* = .643, η_p_^2^ = .01. The three-way interaction between modality relation, response relation, and distractor relation was not significant, *F*(1, 37) = 3.28, *p* = .078, η_p_^2^ = .08; however, a descriptively similar pattern was observed as in the RT analysis. The distractor–response binding effect was by trend only positive in the modality repetition trials, but negative in the modality change trials. For the sake of completeness, the interaction of modality relation and response relation was significant, *F*(1, 37) = 31.36, *p* < .001, η_p_^2^ = .46. None of the other effects were significant, *F*s(1, 37) < 3.02, *p*s > .091.

## Discussion

The present study was designed to specifically analyze the level of representation of irrelevant stimuli. Previous studies found evidence for the representation of distractor stimuli at a conceptual level (e.g., Frings et al., 2013). However, these studies have some crucial shortcomings (e.g., task relevance of the distractor modality; mapping of the distractor stimuli to specific responses) that make a clear-cut interpretation difficult. To this end, we investigated whether distractor-based retrieval can be initiated by the repetition of distractor stimuli at a conceptual and/or perceptual level in a task design that enables an unambiguous conclusion with regard to the level of representation of the distractor stimuli.

In the present study, we implemented a crossmodal distractor–response binding paradigm that allows the repetition and change of conceptual distractor features (i.e., the specific distractor rhythm) independent of the repetition of perceptual distractor features (i.e., the concrete modality). Furthermore, in this paradigm, the distractor modality was never task-relevant. More precisely, the target stimulus was always presented to vision while the distractor stimuli could be presented either to audition or touch. If distractor stimuli are represented at a perceptual level, distractor-based retrieval would only be expected if the perceptual distractor features are repeated—that is, if the distractor modality is repeated from prime to probe. If distractor stimuli are represented at a conceptual level, distractor-based retrieval can be initiated by the repetition of the distractor identity in a different modality—that is, due to a sole repetition of conceptual distractor features.

In the present experiment, we found significant distractor-based retrieval as shown by the interaction of response relation and distractor relation. Importantly, the factor modality relation significantly modulated the interaction of response relation and distractor relation. Distractor-based retrieval was only observed if the modality of the distractor was repeated from prime to probe, but not if the distractor modality changed from prime to probe (see Fig. 1b). This finding fits with the assumption that task-irrelevant distractor stimuli are represented at a perceptual level. The perceptual code of a distractor (i.e., the specific modality) is automatically activated during first encounter and thus is integrated into the event file. If the same perceptual code is then again presented, it can retrieve a previous episode containing the same perceptual code. This pattern was not observed for the conceptual distractor feature. Conceptual features might not be automatically activated and thus are not integrated into the event file and cannot retrieve a previous episode. Consequently, the repetition of perceptual distractor features is indispensable for the initiation of the distractor-based retrieval process. Furthermore, the observed pattern of results contradicts the idea that distractor stimuli are represented at a conceptual level. That is, no evidence for crossmodal distractor-based retrieval was found.

The present results clearly indicate a representation of distractor stimuli at a perceptual level. Yet this does not mean that a distractor stimulus could never be represented at a conceptual level. In fact, previous studies have already shown crossmodal retrieval effects, at least if the stimulus modality is relevant to the task (Frings et al., 2013; Wesslein & Frings, 2020). Indeed, the present finding suggests that the representation of distractor stimuli at a conceptual level is not a general phenomenon that can be observed in every situation, but is dependent on specific task conditions. Certainly, the finding that the retrieval process is dependent on the repetition of the specific percept of the stimulus is in line with the assumption of a direct link between perception and action, independent of semantic processing (e.g., Hommel et al., 2001). Importantly, these conclusions can with certainty only be drawn for the used experimental setup (e.g., the used stimuli and combinations of modality and rhythms). Indeed, previous research in the binding literature found evidence that the same effects and pattern of results can be found with different stimuli and can be transferred to different modalities (e.g., Moeller & Frings, 2011; Moeller et al., 2012). However, further studies with different combinations of modality and rhythm need to be run to ensure that the present findings can be generalized.

It should be noted that we cannot verify whether the stimuli were perceived synchronously or whether there was a temporal order in which stimuli from different modalities were registered. However, this is not problematic for the investigation of the distractor–response binding effect in the present experiment. Previous studies have shown that the integration process is very flexible with regard to temporal factors. More precisely, evidence was found that integration of stimuli takes place inside a binding window of ±500 ms (e.g., Hommel, 2005) and that even the temporal order of stimuli is not decisive for the observation of the distractor–response binding effect (see also Hommel, 2009). Please note that if little asynchronies in the perception of the stimuli in the different modalities existed, this would be in the range of some milliseconds, well within the ±500-ms integration window. Thus, even if some small temporal asynchronies between the stimuli existed, this should not have any effect on the observed binding effect. Furthermore, differences in the perception and processing of auditory and tactile distractor stimuli would only arise in a main effect of modality (which was not observed). In the present study, the focus of interest concerned higher order interactions of response relation and distractor relation in the context of modality repetition and changes. These interactions were not modulated by the particular distractor modality. Thus, possible differences in the perception and processing of the modality had no influence on the observed effects.

In addition, it should be noted that although distractors might be perceived slightly before target onset and thus might have a kind of relevance due to predicting the target presentation, this relevance (if at all) only refers to the information when to respond, but provides no information about how to respond. That is, the distractor stimuli are nevertheless truly task irrelevant, since the distractor stimuli possess no response-relevant target feature and provide no information about the to-be-executed response.

Moreover, the use of rhythms as distractor stimuli has one shortcoming—that is, the distractor stimuli are distinguishable only after a certain time interval (after about 150 ms in the present study). As a result, the information about the sensory modality (i.e., the perceptual distractor feature) might be available earlier in time than the information about the stimulus identity (i.e., the conceptual distractor feature) and thus might have an advantage in influencing response selection processes. As a matter of fact, evidence was found in the present study that the modality per se has an influence on the decision-making process by finding an interaction of response relation and modality relation, showing that the response repetition effect (faster responses if the response is repeated than if the response is changed) is stronger for modality repetition trials in contrast to modality change trials. Importantly, this interaction is further qualified by an interaction of response relation, modality relation, and distractor relation. Thus, although the conceptual distractor feature might be available later in time, this clearly does not prevent an influence of this factor on the response selection process. In addition, it should be noted that various other studies have found an influence of stimulus identity/rhythm on the decision-making process despite using stimuli that only became distinguishable after a certain time interval (e.g., Frings & Spence 2010; Moeller & Frings, 2011; Wesslein et al., 2014).

Furthermore, one could argue that the integration of distractor and target in the present study might not work well since those stimuli were semantically related to a lesser extent than in previous studies that found distractor-based retrieval at a conceptual level (e.g., Frings et al., 2013). However, there is ample evidence from other studies contradicting this assumption. For instance, evidence was found that the integration process is a highly automatic and rather nonselective process (Hommel, 2005). In line with that, previous studies indicated that the distractor–response binding effect is a robust effect that can be replicated with all kinds of stimuli (e.g., words, letters, shapes, faces). Additionally, a study by Giesen et al. (2012) showed that binding effects are observed independent of the relation between the distractor and the target stimulus—that is, the strength of the binding effect does not depend on whether the stimuli are from the same or from different stimulus sets and not on whether the distractor stimuli are neutral or task-related.

The present study fits nicely with research concerning feature integration across modalities (for a recent discussion, see Spence & Frings, 2019). Several studies have already shown that integration is not only possible within modalities (visual: e.g., Frings & Rothermund, 2011; Zmigrod & Hommel, 2010; auditory: e.g., Moeller et al., 2012; Zmigrod & Hommel, 2010; tactile: e.g., Moeller & Frings, 2011; Wesslein et al., 2019) but also that stimuli from different modalities can be integrated in one event file (Zmigrod & Hommel, 2010; Zmigrod et al., 2009; see Zmigrod & Hommel, 2013, for a review). In fact, feature integration across modalities is also observed in the present study by our general finding of distractor–response binding (as the distractors were always presented in a different modality than the target).

From a crossmodal/multisensory perspective, the present study stands in a long line of research showing crossmodal influences of a seemingly irrelevant stimulus in one sensory modality on a task-relevant stimulus in another (e.g., the crossmodal congruency task; for reviews, see Spence, 2020; Spence et al., 2008). In the present study, we took a closer look at the way in which this seemingly irrelevant stimulus is processed. This comes with an interest in recent years about the processing of irrelevant information in truly multisensory settings (e.g., Jensen et al., 2019a; Merz et al., 2019). Interestingly, first evidence indicates that multisensory stimuli are processed at the perceptual level (Jensen et al., 2019b), yet at this point it is way too early to come to general conclusions due to critical differences in the tasks used to investigate information processing across the senses (for a review and discussion, see Merz et al., 2020).

Taken together, using an experimental design in which distractors are completely irrelevant for responding concerning the stimulus–response mapping and even the modality they are presented to, we found no evidence for a conceptually based distractor–response binding effect, at least with the stimuli and experimental setup used here. Previous findings of crossmodal or conceptual binding effects occurred due to attentional weighting or response confounds—but without these confounds, the ‘standard value’ for distractors representations seems to be at a perceptual level.

## Footnotes

1 All analyses were additionally conducted with the two excluded participants. The pattern of significance was the same in the RT analysis. In the error analysis, the three-way interaction of response relation, distractor relation, and modality relation reached significance, *F*(1, 37) = 4.81, *p* = .034, η_p_^2^ = .11, due to the significant negative distractor-response binding effect in the modality change condition, *t*(37) = −2.23, *p* = .032, *d* = 0.35.

2 This pattern is not dependent on the factor target relation—that is, on whether the response repetition included a target repetition or a target change (which are both possible due to the two-to-one mapping of targets and responses), *t*(37) = 1.61, *p* = .116, *d* = 0.26.

3 The analysis was carried out additionally with the variable modality (tactile vs. auditory) in a four-factorial ANOVA. Neither the distractor-response binding effect, *F*(1, 37) < 1, *p* = .615, *η*_p_^2^ < .01, nor the three-way interaction, *F*(1, 37) < 1, *p* = .816, *η*_p_^2^ < .01, was modulated by the respective modality. Furthermore, no main effect of modality was observed, *F*(1, 37) = 2.09, *p* = .156, *η*_p_^2^ = .05.

4 Further analyses indicated that the pattern of results in modality change trials was not dependent on the specific modality, *F*(1, 37) < 1, *p* = .828, η_p_^2^ < .01. The distractor-response binding effect in modality change trials is neither observed if the modality changes from a tactile distractor in the prime to an auditory distractor in the probe, *t*(37) < 1, *p* = .848, *d* = 0.03, nor if the modality changes from an auditory distractor in the prime to a tactile distractor in the probe, *t*(37) < 1, *p* = .581, *d* = 0.09. Furthermore, no evidence was found that the distractor-response binding effect in modality repetition trials was different for auditory and tactile distractors, *F*(1, 37) < 1, *p* = .607, η_p_^2^ = .01, for the three-way interaction of response relation, distractor relation, and modality (auditory vs. tactile) in modality repetition trials.
